# Interspecific Differences in Physiological and Biochemical Traits Drive the Water Stress Tolerance in Young *Morus alba* L. and *Conocarpus erectus* L. Saplings

**DOI:** 10.3390/plants10081615

**Published:** 2021-08-06

**Authors:** Zikria Zafar, Fahad Rasheed, Ahsan Ul Haq, Faridah Hanum Ibrahim, Shazia Afzal, Mohd Nazre, Seemab Akram, Zafar Hussain, Kamziah Abdul Kudus, Muhammad Mohsin, Abdul Qadeer, Zohaib Raza, Waseem Razzaq Khan

**Affiliations:** 1 Department of Forestry & Range Management, University of Agriculture, Faisalabad 38040, Pakistan; zikria.zafar@forst.uni-goettingen.de (Z.Z.); ahsanmalik411@hotmail.com (A.U.H.); zohaib-raza@outlook.com (Z.R.); 2 Department of Forest Genetics and Forest Tree Breeding, University of Göttingen, 37077 Buesgenweg, Germany; 3 Institut Ekosains Borneo, Universiti Putra Malaysia Kampus Bintulu, Bintulu 97008, Malaysia; f_hanum@upm.edu.my; 4 Department of Forestry, University of Sargodha, Sargodha 40100, Pakistan; shazia.afzal@uos.edu.pk; 5 Department of Forestry Science and Biodiversity, Faculty of Forestry and Environment, Universiti Putra Malaysia, Sri Serdang 43400, Malaysia; nazre@upm.edu.my (M.N.); kamziah@upm.edu.my (K.A.K.); 6 Department of Biology, Faculty of Science, Universiti Putra Malaysia, Sri Serdang 43400, Malaysia; seemabakram@ymail.com; 7 Department of Forestry, Range Management and Wildlife, Bahauddin Zakariya University, Multan 60000, Pakistan; zafarfrw@bzu.edu.pk; 8 School of Forest Sciences, University of Eastern Finland, 80100 Joensuu, Finland; muham@uef.fi; 9 Institute of Soil and Environmental Sciences, University of Agriculture, Faisalabad 38040, Pakistan; aqkhan2437@gmail.com

**Keywords:** drought stress, mulberry, leaf gas exchange, water use efficiency, osmolytes accumulation

## Abstract

Mitigating climate change requires the identification of tree species that can tolerate water stress with fewer negative impacts on plant productivity. Therefore, the study aimed to evaluate the water stress tolerance of young saplings of *C. erectus* and *M. alba* under three soil water deficit treatments (control, CK, 90% field capacity, FC, medium stress MS, 60% FC and high stress, HS, 30% FC) under controlled conditions. Results showed that leaf and stem dry weight decreased significantly in both species under MS and HS. However, root dry weight and root/shoot ratio increased, and total dry weight remained similar to CK under MS in *C. erectus* saplings. Stomatal conductance, CO_2_ assimilation rate decreased, and intrinsic water use efficiency increased significantly in both species under MS and HS treatments. The concentration of hydrogen peroxide, superoxide radical, malondialdehyde and electrolyte leakage increased in both the species under soil water deficit but was highest in *M. alba*. The concentration of antioxidative enzymes like superoxide dismutase, peroxidase, catalase, and ascorbate peroxidase also increased in both species under MS and HS but was highest in *C. erectus*. Therefore, results suggest that *C. erectus* saplings depicted a better tolerance to MS due to an effective antioxidative enzyme system.

## 1. Introduction

Globally, drought is considered to be the most common abiotic stress and unpredictable constraint that restricts plant growth and productivity under climate change [1,2]. Pakistan is among the most vulnerable countries to climate change, as the agricultural sector has been subjected to biotic and abiotic pressures in recent years due to climate change [3]. Additionally, Pakistan is one of the countries confronting the severe scarcity of irrigation water as the country diverts 90% of fresh water for agriculture sector to sustainable agriculture production [4]. Approximately 80% of the agriculture land in Pakistan receives canal water, which is also expected to diminish by 32% by 2025 [5]. During the last decade, along with the agricultural sector, tree survival and forest productivity have decreased significantly due to severe water stress under climate change [6,7]. The increasing temperatures and frequent weather extremes like prolonged and severe droughts have rendered forests vulnerable [8]. Thus, increased water scarcity has stirred up the interest to turn every conceivable water drop into plant productivity [9]. Therefore, there is an urgent demand to initiate new strategies where drought tolerant species may be promoted to sustain productivity and curtail water requirements [10]. In this case, tree species that can withstand water stress with minimal productivity loss can be a solution to ensure the sustainable productivity and economic stability of resource poor farmers of the developing countries.

Water scarcity results in the reduction of cell water potential, disruption in cellular activities, and decrease in photosynthesis. Previously, numerous experiments to evaluate the water stress tolerance were carried out on various tree species, such as *Syzygium cumini*, *Salix tetrasperma*, *Acacia modesta*, *Ficus benjamina* and *Zizipus jujube* [11,12,13,14]. These studies have shown that plant sensitivity to drought is a complex phenomenon and contingent on various factors, such as growth and genetic potential, duration and severity of stress, distresses in enzyme activities, and ion balancing that eventually causes yield diminution [15]. Increase in root dry weight, sustaining net CO_2_ assimilation rate and increasing water use efficiency, have been regarded as mechanisms of sustaining growth and deferring the negative of water stress [11,12,13]. Zafar [13] and Rasheed [14] have reported several physiological adjustments in plants that were subjected to water stress. The studies reported that cytoplasmic membrane structure becomes damaged due to dehydration of leaves caused by extravasation of water in the intracellular spaces that results in enhanced contents of malondialdehyde (MDA) and reactive oxygen species [ROS] [16,17]. As a result, the plants tend to increase the activity of antioxidants like superoxide dismutase (SOD), peroxidase (POD), catalase (CAT) and ascorbate peroxidase (APX), to regulate the overproduction of ROS and to protect plant cells from oxidative damage [18,19]. Furthermore, an increase in the production of proline and other osmolytes has also been observed to sustain cell turgor that further enables the cells to mutilate less due to water deficit [20,21]. Therefore, changes in physiological traits and the production of various oxidants and antioxidants reflect the degree of water stress damage and are consequently useful for assessing water deficit tolerance of plants [22,23].

*Morus alba* L. (Mulberry, *M. alba*) belonging to the family *Moraceae* is a perennial woody plant, that has a high economic and ecological importance globally [24]. There are certain remarkable features which have made mulberry a candidate species to be planted under semiarid regions like high biomass production, fruit production, dynamic plant-microbe interactions, nutritional and medicinal significance, as well as endogenous abiotic stress tolerance involving cold, waterlogging, drought, salinity, and heavy metal ions [25]. *Conocarpus erectus* L. (*C. erectus*) belong to the family *Combretaceae*. The species is native to riverine areas of Somalia, Djibouti, Yemen, horn of Africa, Arabian Peninsula, and South Asia [26]. *C. erectus* has also been recognized in several countries as a reliable candidate to withstand different harsh environments, including high temperature, salinity and drought stress of the tropical and sub-tropical regions [27]. Due to its fast growth, long hairy and deep roots, high nutrients and metals uptake and large biomass production potential, this species can also be important to revegetate the arid to semi-arid region [26]. However, evidencing the drought tolerance mechanism of these species is important before recommending these species in revegetation programs. Consequently, comparing the drought tolerance mechanism of *M. alba* and *C. erectus* under water limited regimes can be very important. Therefore, the study aimed to examine the effects of water deficit on *M. alba* and *C. erectus* and to examine the interplay of various physiological, morphological and biochemical parameters in regulating the tolerance mechanism of these two species.

## 2. Materials and Methods

### 2.1. Plant Material and Growth Condition

A pot experiment was conducted in a greenhouse at forestry nursery (31°26′ N, 73°06′), Department of Forestry & Range Management, University of Agriculture Faisalabad, Pakistan. The temperature in the green house (25 ± 5 °C) and the relative humidity of 55 ± 5% was maintained. Furthermore, 3–4 months old, healthy, undamaged and uniform size saplings of *C. erectus* and *M. alba* were selected from a forestry nursery. Every pot (25 × 30 cm and weight 260 ± 5 g) was filled with 8 kg mixture of sandy loam soil and farmyard manure (3:1) and electricity conductivity (2.5 d Sm^−1^). To optimize the nutrient deficiency, NPK fertilizer (15% N; 5% P_2_O_5_; 5% K_2_O) was added at a rate of 5 g/kg of soil.

### 2.2. Water Deficit Treatments

Three various levels of water deficit treatments were maintained at control (CK) = 90% of field capacity FC, medium stress (MS) = 60% FC, high stress (HS) = 30% FC following the method of Rasheed et al. [28]. Every pot was watered back daily to the reference weight by weighing the pots and adding the amount of water lost during evapo-transpiration. A total of 60 healthy and uniform sized saplings of *C. erectus* and *M. alba* (10 saplings per treatments) were selected for this experiment and were randomly assigned either to control (CK), medium water stress (MS), and high-water stress (HS). The experiment was continued for 90 days.

### 2.3. Growth and Biomass Traits Measurements

The following growth parameters, such as plant height (cm), stem diameter (mm) and number of leaves were measured during the experiment. After 90 days, all plants are uprooted and divided into different sections (leaves, stem, and root) were separately placed in paper bags and dried at 70 °C for 72 h in oven (DGH-9202 Series, Thermal Electric Thermostat drying oven, HC, China). Then, the dry weight of all the plant section leaves, stem and roots dry weight and root/shoot ratio (R:S) as the ratio of root dry weight and shoot dry weight was calculated [11].

### 2.4. Chlorophyll a, b, Carotenoid Contents and Leaf Gas Exchange Measurements

Healthy and undamaged leaves were collected at the end of the experiment from each saplings and chlorophyll *a*, *b* and carotenoids contents were estimated following the methods of [29]. Leaf gas exchange attributes were determined before the final harvesting of the experiment. Healthy and mature leaves were selected and different traits like CO_2_ assimilation rate (Ar, μmol CO_2_ m^−2^ s^−1^) and stomatal conductance (g_s_, mol m^−2^ s^−1^) were determined by using the portable infrared gas analyzer (CIRAS-3, Amesbury, MA, USA). All measurements were determined between 10:00 am to 12:00 pm. Intrinsic water use efficiency (WUE_i_, μmol mol^−1^) was determined as the ratio of net CO_2_ assimilation rate and stomatal conductance [28].

### 2.5. Determination of Proline, Soluble Sugar, Total Phenolic Content and Total Soluble Protein

The proline content in leaf samples was measured by using the ninhydrine method demonstrated by Bates [30]. Total phenolic content was calculated following the method of Ainsworth and Gillespie [31]. Total soluble protein was estimated by following the Bradford method [32] and total soluble sugar was estimated by using the Anthrone method demonstrated by Yemm and Willis [33].

### 2.6. Determination of Malondialdehyde Contents (MDA) and Electrolyte Leakage (EL %)

Lipid peroxidation was measured in the form of malondialdehyde (MDA) content as the method demonstrated by Hodges [34]. Electrolyte leakage (EL %) was estimated as demonstrated by Nayyar [35]. Moreover, 0.2 g samples were rinsed in tubes with 20 mL of deionized water and the tubes were placed in a water bath at 32 °C. After 1290 min, the initial electrical conductivity (ECi) was calculated with a digital EC meter (Model DJS-1C Model DJS-1C; Shanghai Analytical Instrument Co., Shanghai, China). Then, all the samples were heated at 100 °C for 20 min, cooled at room temperature and final electrical conductivity (ECf) was measured.

(EL %) = (ECi/ECf) × 100

### 2.7. Production of ROS and Antioxidant Enzyme Activity

The production of hydrogen peroxide (H_2_O_2_) in the leaf samples was calculated as demonstrated by Velikova [36], and the superoxide radical (O_2_^–^) was estimated according to the Bai [37]. Different antioxidative enzyme activity, such as superoxide dismutase (SOD), was estimated by the photochemical reduction of NBT (nitroblue tetrazolium) as methods demonstrated by Bayer [38]. Peroxidase (POD) enzyme activity was measured as a method demonstrated by Maehly and Chance [39]. The catalase (CAT) activity was estimated according to the Knörzer [40] and ascorbate peroxidase (APX) was determined according to the Nakano and Asada [41].

### 2.8. Data Analysis

All the data corresponding to growth attributes, biomass production, chlorophyll *a*, *b*, carotenoids contents, osmolytes accumulation, oxidants and antioxidants enzyme concentration were analyzed using the two-way ANOVA for species effect (S effect), treatments (T effect) and their interaction (S × T effect). A significant difference was compared with their control using Dunnett’s test. All means are presented with their standard error (±SE) and all tests were taken to be significant at *p* < 0.05.

## 3. Results

### 3.1. Effect of Soil Water Deficit on Growth and Dry Weight Production

In *C. erectus*, the mean plant height and stem diameter increased significantly (5.0% and 5.7%) under MS but decreased significantly under HS (14% and 25%), while root: shoot ratio (R:S ratio) was increased significantly by 27% and 94% under both MS and HS as compared to CK (Table 1).

**Figure 1:**
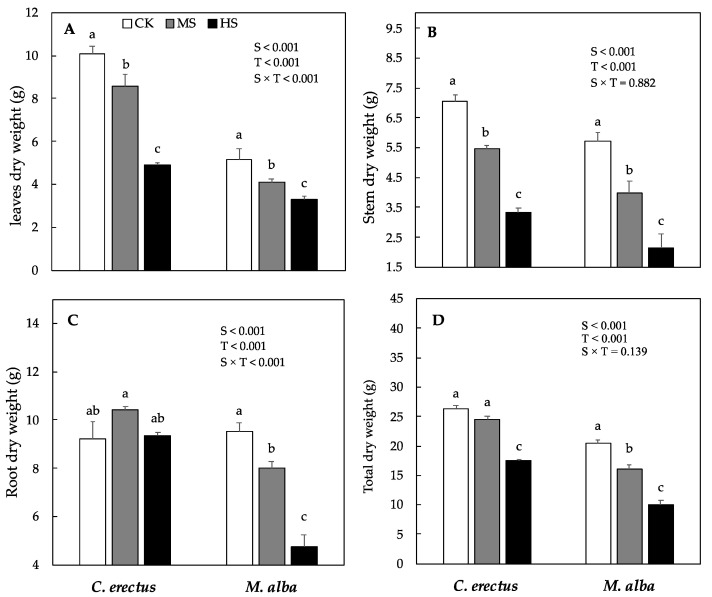
Mean dry weight production in leaves dry weight (**A**), stem dry weight (**B**), root dry weight (**C**) and total dry weight (**D**) under control (CK), Medium (MS) and high (HS) water deficit treatments in *C. erectus* and *M. alba*. All the attributes were tested using a two-way ANOVA for species effect (S-effect), treatment (T-effect) and interaction (S × T) effects. All values represent the means (±SE) of species in various combinations. Small letters set in bold represent significant difference within each species tested using *Dunnett’s* test. All the tests were taken significant at *p* < 0.005.

In *M. alba*, mean plant height significantly decreased by 20% and 37% under MS and HS and stem diameter remained similar to CK under MS, but decreased by 21% under HS as compared to CK. R:S ratio increased significantly under MS and remained similar to CK under HS (Table 1). Leaf, stem, root and total dry weight differed significantly among species and treatments. In *C. erectus* saplings, leaf and stem dry weight decreased significantly under both MS and HS (14.8 and 22; 51% and 52.7%, respectively; Figure 1A,B). However, the root dry weight increased significantly (13%) under MS and remains similar to control under HS (Figure 1C). Consequently, total dry weight remains similar under MS and decreased by (33%) under HS respectively, as compared to CK (Figure 1D). In *M. alba* saplings, a significant decrease was observed in leaf, stem, root and total dry weight under MS, which further decreased by 36%, 62%, 50%, and 50% under HS as compared to CK, respectively (Figure 1).

### 3.2. Effect of Soil Water Deficit on Chlorophyll a, b and Carotenoid Contents

Chlorophyll *a*, *b* (Chl *a*, *b*) and carotenoids contents varied significantly among species, as well as across the treatments. In *C. erectus* saplings, the Chl *a*, *b* and carotenoid content decreased significantly under both MS and HS treatments. However, the highest reduction in Chl *a*, *b* and carotenoid content (44%, 44% and 27%) was found under HS as compared to CK, respectively (Table 2). Similarly, in *M. alba* the Chl *a*, *b* and carotenoid content decreased significantly under both MS and HS. However, the highest reduction in Chl *a*, *b* and carotenoid of 60%, 55% and 23% respectively, was found under HS treatments as compared to CK.

### 3.3. Effect of Soil Water Deficit on Production of Proline, Soluble Sugar, Total Phenolic Content and Soluble Protein

In both the species *C. erectus* and *M. alba*, proline, soluble sugar, soluble protein and total phenolic varied significantly among both species and treatments. In *C. erectus* saplings, proline, soluble sugar, soluble protein and total phenolic content increased significantly under both MS and HS treatment respectively, however the highest increase was found under HS (71%, 38%, 19% and 74%, respectively) as compared to CK (Table 2). Similarly, in *M. alba* saplings proline, soluble sugar, soluble protein and total phenolic content also increased significantly under both MS and HS treatments respectively, and the highest increase was evidenced under HS (33%, 13%, 31% and 41%, respectively), as compared to CK.

### 3.4. Effect of Soil Water Deficit on Gas Exchange Attributes

**Figure 2:**
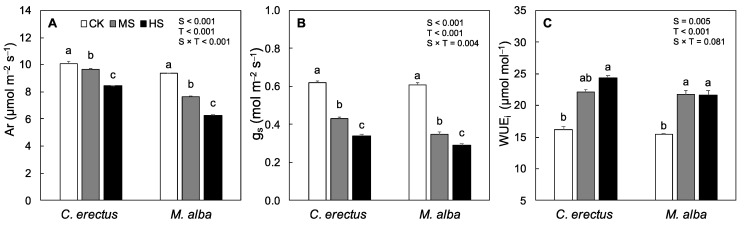
Means values of various gas exchange parameters such as CO_2_ assimilation rate (**A**), stomatal conductance (**B**), intrinsic water use efficiency (**C**) under control (CK), Medium (MS) and high (HS) water deficit treatments in *C. erectus* and *M. alba*. All the attributes were tested using a two-way ANOVA for species effect (S-effect), treatment (T-effect) and interaction (S × T) effects. All values represent the means (±SE) of species in various combinations. Small letters set in bold represent significant difference within each species tested using Dunnett’s test. All the tests were taken to be significant at *p* < 0.005.

The CO_2_ assimilation rate and stomatal conductance varied significantly across the species, as well as treatments. In *C. erectus* saplings, stomatal conductance and CO_2_ assimilation rate decreased significantly under both MS and HS treatments, respectively. However, the highest reduction (16% and 45%) was found under HS (Figure 2A,B). In *M. alba* saplings, the CO_2_ assimilation rate and stomatal conductance decreased significantly (18% and 42%) under MS and (33% and 52%) HS treatments, as compared to CK. Intrinsic water use efficiency (WUE_i_) also varied significantly between the species, as well as across the treatments. In *C. erectus,* WUE_i_ progressively increased in MS and HS (~50%). In *M. alba* saplings, WUE_i_ increased under MS (40%) and no further increase was evidenced under, as compared to CK (Figure 2C).

### 3.5. Effect of Soil Water Deficit on the Production of Reactive Oxygen Species, Malondialdehyde (MDA) and Electrolyte Leakage

**Figure 3:**
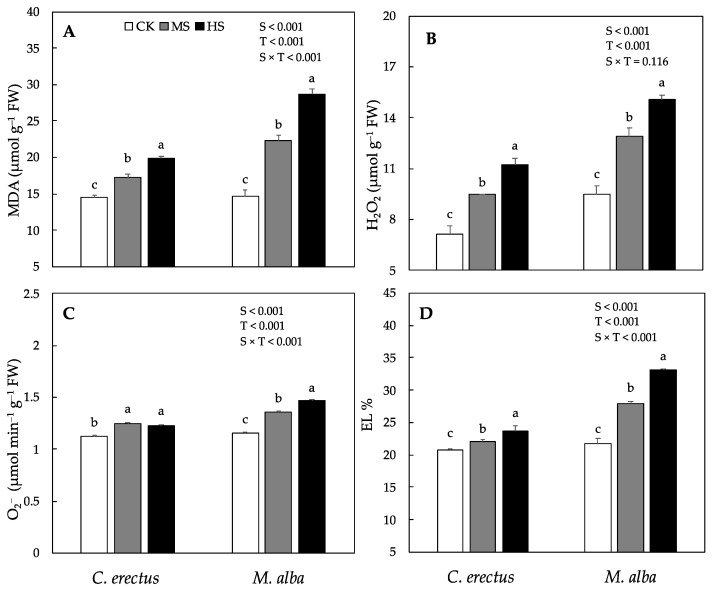
Production of various oxidants, such as hydrogen peroxide, H_2_O_2_ (**A**) and superoxide radical, O_2_^−^ (**B**), along with malondialdehyde contents, MDA(**C**) and electrolyte leakage, EL% (**D**) under control (CK), Medium (MS) and high (HS) water deficit treatments in *C. erectus* and *M. alba*. All the attributes were tested using a two-way ANOVA for species effect (S-effect), treatment (T-effect) and interaction (S × T) effects. All values represent the means (±SE) of species in various combinations. Small letters set in bold represent significant difference within each species tested using *Dunnett’s* test. All the tests were taken to be significant at *p* < 0.005.

The Production of ROS (reactive oxygen species) such as hydrogen peroxide (H_2_O_2_) and superoxide radical (O_2_^−^) along with malondialdehyde (MDA) and electrolyte leakage (EL%) varied significantly between the species as well as the treatments. In *C. erectus* saplings the H_2_O_2_, O_2_^−^, MDA and EL % increased significantly by 32%, 11%, 19% and 6%, respectively under MS, and by 56%, 9%, 37% and 14%, respectively under HS, compared to CK (Figure 3). Similarly, in *M. alba* saplings H_2_O_2_, O_2_^−^, MDA and EL% also increased significantly under MS and HS treatments, however, the increase was highest under HS.

### 3.6. Effect of Soil Water Deficit on the Antioxidant Enzyme Activity

**Figure 4:**
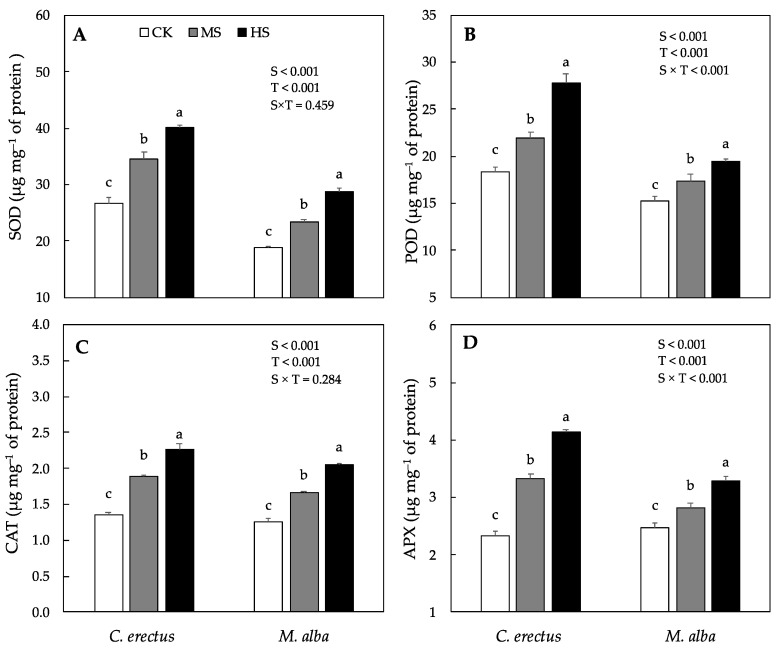
Mean activity various antioxidants enzyme concentration (**A**) superoxide dismutase, SOD, (**B**) peroxidase, POD, (**C**) catalase, CAT and (**D**) ascorbate peroxidase, APX under control (CK), Medium (MS) and high (HS) water deficit treatments in *C. erectus* and *M. alba*. All the attributes were tested using a two-way ANOVA for species effect (S-effect), treatment (T-effect) and interaction (S × T) effects. All values represent the means (±SE) of species in various combinations. Small letters set in bold represent a significant difference within each species tested using Dunnett’s test. All the tests were taken significant at *p* < 0.005.

The activity of antioxidant enzymes, such as superoxide dismutase (SOD), peroxidase (POD), catalase (CAT), and ascorbate peroxidase (APX), varied significantly between the species as well as across the treatments. In *C. erectus* saplings, the activity of SOD, POD, CAT and APX progressively increased under both MS and HS treatments, respectively. The highest increase was found under HS (50%, 52%, 67% and 77%) as compared to CK. Similarly, in *M. alba* saplings, the activity of SOD, POD, CAT and APX also increased significantly under both MS and HS treatments respectively, with the highest increase was found under HS (52%, 28%, 62% and 33%) respectively, as compared to CK (Figure 4).

## 4. Discussion

Water deficit is one of the most limiting factors for plant growth and productivity. Under water deficit environment, the most obvious response is the decrease in morphological and physiological traits, such as growth and photosynthesis rate [29,42]. In the present study, *C. erectus* and *M. alba* demonstrated a substantial decrease in the growth parameters, such as plant height, stem diameter and dry weight (leaves, stem, and total) under HS treatment (Table 1 and Figure 1). However, the decrease was the lowest in *C. erectus* and was the highest in *M. alba* saplings. These findings are in accordance with the previous studies on various tree species (*Ficus*, *Ziziphus jujube*, *Syzygium cumini* and *Eucalyptus globulus*), where a significant reduction on growth and biomass production has been reported under water deficit condition [11,12,13,43,44]. The reduction in growth and biomass production under the limited supply of water environment is related to low turgor pressure, the reduction of cell division and the shrinkage of cell volume, which is mediated by the low water and nutrient uptake due to the less diffusion of water from soil matrix into roots [11,12]. Furthermore, in *C. erectus* saplings, the plant height, stem diameter and total dry weight remain similar to control under MS. The negative impact of water stress on plant growth is species-specific and also depends on the duration and intensity of stress. These results are in line with the previous studies on *Syzygium cumini* and *Zizipus jujube*, where no negative impact on growth and plant morphology was evidenced under medium water stress conditions [12,13]. Root morphology is one of the important factors that determine the ability of a species to survive under a limited supply of water [45,46]. Generally, drought tolerant species demonstrate an increase in root growth under water stress condition that helps the plant to absorb water from a larger volume of soil, and thus helps in delaying water stress-induced negative changes [13,47]. In the present study, the R:S ratio increased in both species under MS. However, an increase in R:S ratio was highest in *C. erectus* saplings (Table 1), which shows that *C. erectus* is better adapted for medium water stress conditions than *M. alba*. These results are in agreement with the previous studies, where the increase in the R:S ratio in water deficit has been related to the increased drought stress tolerance in *Conocarpus erectus* L., *Ficus benjamina*, *Ziziphus jujuba* and *Syzygium cumini* L. [11,12,13]. Based on our results, it can be concluded that *C. erectus* showed a better adaptability to medium water stress as compared to *M. alba*, which was due to a significant increase in root development.

The reduction in chlorophyll content is a common response to the abiotic stresses [47,48] that results in reduction in photosynthesis and assimilation rate under soil water deficit environment [49]. In this study, stomatal conductance and Chl *a* and *b* decreased significantly, along with carotenoid content (Table 2). However, it has been reported in the previous studies that photosynthetic rate is maintained even under high water stress and low leaf water contents in species like *Arabidopsis*, *Lupinus albus*, *Helianthus annuus*, *Vitis vinifera* and *Eucalyptus globulus*, *Populus euphratica* [48,50]. Moreover, Flexas [51] demonstrated that mesophilic components like Rubisc, are particularly resilient and maintain their activity, even under high water stress, e.g., even when relative leaf water content drops to 50% and stomata are 75% closed. Therefore, it can be assumed that an observed decrease in CO_2_ assimilation rate under MS and HS was related to decreased stomatal conductance. Another aspect that can influence the overall fluctuations of CO_2_ assimilation rate are the chlorophyll contents that influence the CO_2_ assimilation rate of the plant. Previous studies have related the decrease in the Chl contents to the decrease in CO_2_ assimilation rate [43]. Reduction in chlorophyll contents under water deficit condition is mostly linked to the chloroplasts damage, due to the overproduction of reactive oxygen species [51]. In this study, Chl *a* and *b*, along with carotenoid contents, decreased under both MS and HS. Therefore, the overall decrease in the CO_2_ assimilation rate under MS and HS can be related to the overall decrease in stomatal conductance and Chl *a* and *b* (Table 2 and Figure 2). Many previous studies have reported similar results where the Chl content together with stomatal conductance were found responsible for the reduction in CO_2_ assimilation rate under water deficit treatments [11,43,47]. Intrinsic water use efficiency (WUE_i_), which is the ratio between CO_2_ assimilation rate and stomatal conductance [28], increased significantly under MS and HS treatments in *C. erectus* and *M. alba* saplings (Figure 2). The results are in line with the previous study, where increases in WUE_i_ have been observed under water deficit treatments respectively, and the decrease in stomatal conductance is much more responsive to the fluctuation of soil moisture than the CO_2_ assimilation rate of [11,28,47]. Therefore, it can be concluded that a decrease in CO_2_ assimilation rate was related to the decrease in Chl *a* and *b* contents under MS and to both Chl *a*, *b* and stomatal conductance under HS.

Proline and soluble sugar are two of the significant elements in the defense system or enhancing the drought stress tolerance in plants [52,53]. In the present experiment, proline and soluble sugar increased significantly in *C. erectus* and *M. alba* saplings under MS and HS, respectively (Table 2). However, the highest concentration of proline and soluble sugar was observed in *C. erectus* saplings. These results are in line with the previous studies where the proline and soluble sugar increased in black poplars, mulberry, olive, *Conocarpus*, *Salix* and *Acacia* plants under a limited supply of water [14,24,54,55]. An increase in proline and soluble sugar content plays a key role in decreasing the osmotic potential, improving the physiological, growth processes and maintaining the water absorption under water deficit condition [56,57]. The phenolic compounds are also important secondary metabolites that play a significant role in increasing plant tolerance under stressful environments [58]. Previous studies have reported that plants up-regulate the expression of phenolics-synthesizing enzymes, such as phenylalanine ammonia-lyase, that result in the increased production of phenolic compounds that may also act as the antioxidants under stressful conditions [59]. In the present study, the production of phenolic compounds increased significantly under water deficit treatment in both *C. erectus* and *M. alba* saplings (Table 2). These results are in line with the previous studies where an increase in phenolic compound and soluble protein has been observed in *Salix* and *Acacia* [47], *Portulaca oleracea* [60], *Eucalyptus globulus* [61] *Syzygium cumini* [13], under water stress conditions.

The balance between the production of oxidants and antioxidants plays a key role in determining tolerance status of species to different types of abiotic stress. Different studies have shown that water stress environment induces the production of reactive oxygen species, such as hydrogen peroxide (H_2_O_2_), superoxide radical (O_2_^−^) and singlet oxygen (^1^O_2_) [47,62]. In this study, the production of H_2_O_2_ and O_2_^−^ increased significantly in *C. erectus* and *M. alba* saplings under MS and HS treatments (Figure 3). These results are similar to the previous findings where an increase in the production of H_2_O_2_ and O_2_^−^ have been reported in *Salix*, *Acacia*, *Portulaca*, *Quercus* and other woody tree species under water deficit treatments [47,60,63,64]. Various studies have elaborated that an increase in the accumulation of ROS disrupts the redox balance and this ultimately decreases the productivity of plants under limited supply of water [65]. Our study also showed that the significant increase in ROS and resulted in a significant decrease in dry weight production in *C. erectus* and *M. alba* saplings under HS. It has been reported that the cell membrane is highly sensitive to different abiotic stress and increase in the concentration of malondialdehyde (MDA) contents and electrolyte leakage (EL%) indicates the extent of cellular damage [47,60]. In this study, we noticed a significant increase in MDA content and EL% in both *C. erectus* and *M. alba* under MS and HS (Figure 2). These findings are in line with previous studies where a significant increase in EL% and MDA contents in *Cassia occidentalis*, *Conocarpus erectus*, *Acacia modesta*, *Salix tetrasperma*, *Quercus brantii*, *Portulaca oleracea* have been reported under a limited supply of water [47,60,64].

In order to cope with the increased production of ROS, such as H_2_O_2_ and O_2_^−^, plants have a strong defense system of antioxidant enzymes (SOD, POD, CAT and APX) and some non-enzymatic antioxidants (ascorbic acid, anthocyanin and proline) [61]. In our experiment, the activity of antioxidant enzymes such as SOD, POD, CAT and APX increased significantly in both species under MS and HS treatments. However, the highest increase was found in *C. erectus* compared to *M. alba* saplings (Figure 4). Similar results have been reported in the previous studies where the activity of antioxidative enzymes increases significantly in *Acacia modesta*, *Salix tetrasperma*, *Portulaca oleracea*, *Robinia pseudoacacia* and olive cultivars under soil water deficit treatments [20,47,53,60,64]. An increase in the antioxidant enzyme activity is also related to the improved drought tolerance in mulberry [24], *P. vulgaris* [66] and olive plants [20]. In this regard, SOD is the first line of defense against the overproduction of ROS [67], which plays an important role in lowering the production of reactive oxygen species [68] by the dismutation of O_2_^−^ into H_2_O_2_. The H_2_O_2_ are then further eliminated by CAT, POD and other antioxidant enzymes. Studies have demonstrated that CAT is usually present in the peroxisome and is implicated in balancing out the increased concentration of H_2_O_2_, while CAT converts the H_2_O_2_ to H_2_O in the plant cell. Therefore, it can be concluded that the highly effective antioxidative defense mechanism may have played a vital role in sustaining the total dry weight production and enhancing water stress tolerance in *C. erectus* under MS as compared to *M. alba* saplings.

## 5. Conclusions

Results showed that soil water deficit had a negative effect on both *C. erectus* and *M. alba* saplings. However, the highest reduction in morphological traits (leaves and stem dry weight production) was evidenced in *M. alba* saplings, as compared to *C. erectus*. Interestingly, root dry weight production, R:S ratio and total dry weight were significantly increased in *C. erects* saplings under MS treatments. Higher tolerance shown by *C. erectus* under MS can be linked to the increased root growth and maintenance of CO_2_ assimilation rate and increase in the concentration of osmolytes, which could have increased the water absorption and retention capability of this species as compared to *M. alba*. Finally, the production of H_2_O_2_, O_2_^−^, MDA and EL % increased significantly under both MS and HS treatments, but the highest increase was found in *M. alba* saplings showing higher vulnerability of this species even to a moderate soil water deficit. On the other hand, the highest increase in the activity of antioxidant enzymes (SOD, POD, CAT and APX) was found in *C. erectus* that could have been more effective in mitigating the increased production of oxidants in *C. erectus* saplings as compared to *M. alba*. Therefore, it can be concluded that the *C. erectus* saplings showed higher tolerance to medium soil water deficit treatments than the *M. alba* saplings, which was related to the consistent CO_2_ assimilation rate, increased osmolyte accumulation and the increased of antioxidative enzyme activity.

## Figures and Tables

**Table 1:** Effect of water deficit on various growth attributes like plant height, stem diameter and root: shoot ratio (R:S ratio) in *C. erectus* and *M. alba*. The traits were tested using a two-way ANOVA for species effect (S-effect), treatment (T-effect) and interaction (S × T) effects. Values represent the means (±SE) in various treatments. Small letters represent significant differences within each species tested using Dunnett’s test. Test were taken to be significant at *p* < 0.005.

	Traits	Plant Height (cm)	Stem Diameter (mm)	R:S Ratio
	CK	65.5 ± 1.85 ^a^	5.03 ± 0.22 ^ab^	0.58 ± 0.04 ^b^
*C. erectus*	MS	68.8 ± 1.70 ^a^	5.32 ± 0.19 ^a^	0.74 ± 0.03 ^ab^
	HS	56.3 ± 1.85 ^b^	3.73 ± 0.09 ^b^	1.13 ± 0.03 ^a^
	CK	62.9 ± 0.93 ^a^	4.67 ± 0.55 ^a^	0.88 ± 0.05 ^ab^
*M. alba*	MS	49.7 ± 0.60 ^b^	4.58 ± 0.25 ^a^	0.99 ± 0.02 ^a^
	HS	39.1 ± 1.70 ^c^	3.67 ± 0.30 ^b^	0.91 ± 0.22 ^ab^
S-effect		*p* < 0.001	*p* = 0.532	*p* = 0.143
T-effect		*p* < 0.001	*p* < 0.001	*p* = 0.014
S × T effect		*p* < 0.001	*p* = 0.522	*p* = 0.017

**Table 2:** Effect of water deficit on Chl *a*, *b*, carotenoid, proline, soluble sugar, soluble protein and total phenolic content under various water deficit treatments respectively in *C. erectus* and *M. alba*. All the traits were tested using a two-way ANOVA for species effect (S-effect), treatment (T-effect) and interaction (S × T) effects. All values represent the means (±SE) of species in various combinations. Small letters represent significant difference within each species tested using *Dunnett’s* test. All the tests were taken significant at *p* < 0.005.

	Traits	Chl *a* (mg g^−1^ FW)	Chl *b* (mg g^−1^ FW)	Carotenoid (mg g^−1^ FW)	Proline (μg g^−1^ FW)	Soluble Sugar (mg g^−1^ FW)	Soluble Protein (mg g^−1^ FW)	Total Phenolic Contents (mg g^−1^ FW)
	CK	3.30 ± 0.07 ^a^	3.05 ± 0.02 ^a^	0.85 ± 0.01 ^a^	17.4 ± 0.63 ^c^	66.5 ± 0.69 ^c^	25.4 ± 0.49 ^c^	1.22 ± 0.02 ^c^
*C. erectus*	MS	2.72 ± 0.05 ^b^	2.16 ± 0.04 ^b^	0.72 ± 0.01 ^b^	25.5 ± 0.94 ^b^	74.9 ± 1.12 ^b^	27.3 ± 0.30 ^b^	1.68 ± 0.05 ^b^
	HS	1.83 ± 0.05 ^c^	1.70 ± 0.05 ^c^	0.62 ± 0.03 ^c^	29.9 ± 0.48 ^a^	91.8 ± 1.00 ^a^	30.4 ± 0.63 ^a^	2.13 ± 0.02 ^a^
	CK	2.53 ± 0.03 ^a^	2.18 ± 0.06 ^a^	0.80 ± 0.02 ^a^	17.4 ± 0.38 ^c^	71.6 ± 0.89 ^b^	25.4 ± 0.41 ^c^	1.39 ± 0.05 ^c^
*M. alba*	MS	1.87 ± 0.02 ^b^	1.61 ± 0.15 ^b^	0.68 ± 0.00 ^b^	19.3 ± 0.44 ^b^	79.7 ± 0.42 ^a^	29.7 ± 0.54 ^b^	1.61 ± 0.01 ^b^
	HS	1.01 ± 0.02 ^c^	0.98 ± 0.03 ^c^	0.61 ± 0.02 ^c^	23.3 ± 0.34 ^a^	81.1 ± 0.40 ^a^	33.4 ± 0.16 ^a^	1.90 ± 0.02 ^a^
S-effect		*p* < 0.001	*p* < 0.001	*p* = 0.029	*p* < 0.001	*p* = 0.708	*p* < 0.001	*p* = 0.132
T-effect		*p* < 0.001	*p* < 0.001	*p* < 0.001	*p* < 0.001	*p* < 0.001	*p* < 0.001	*p* < 0.001
S × T effect		*p* = 0.714	*p* = 0.131	*p* = 0.545	*p* = 0.004	*p* < 0.001	*p* = 0.072	*p* < 0.001

## Data Availability

Data available on request from the corresponding author.
